# Characteristics of Indium Tin Oxide (ITO) Nanoparticles Recovered by Lift-off Method from TFT-LCD Panel Scraps

**DOI:** 10.3390/ma7127662

**Published:** 2014-11-27

**Authors:** Dongchul Choi, Sung-Jei Hong, Yongkeun Son

**Affiliations:** 1 Department of Chemistry and BK21 PLUS School of HRD Center for Creative Convergence Chemical Science, Sungkyunkwan University, Suwon 440-746, Korea; E-Mail: cdc777@skku.edu; 2 Display Components and Materials Research Center, Korea Electronics Technology Institute, Seongnam 463-816, Korea

**Keywords:** recovery, indium-tin-oxide (ITO), nanoparticle, thin film transistor-liquid crystal display (TFT-LCD) panel, scrap

## Abstract

In this study, indium-tin-oxide (ITO) nanoparticles were simply recovered from the thin film transistor-liquid crystal display (TFT-LCD) panel scraps by means of lift-off method. This can be done by dissolving color filter (CF) layer which is located between ITO layer and glass substrate. In this way the ITO layer was easily lifted off the glass substrate of the panel scrap without panel crushing. Over 90% of the ITO on the TFT-LCD panel was recovered by using this method. After separating, the ITO was obtained as particle form and their characteristics were investigated. The recovered product appeared as aggregates of particles less than 100 nm in size. The weight ratio of In/Sn is very close to 91/9. XRD analysis showed that the ITO nanoparticles have well crystallized structures with (222) preferred orientation even after recovery. The method described in this paper could be applied to the industrial recovery business for large size LCD scraps from TV easily without crushing the glass substrate.

## 1. Introduction

**Figure 1:**
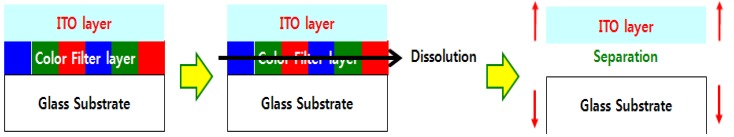
Lift-off method for recovering indium-tin-oxide (ITO) as nanoparticles from glass substrate of thin film transistor-liquid crystal display (TFT-LCD) scraps.

Thin film transistor-liquid crystal display (TFT-LCD) has been used as a most popular flat panel display. As the TFT-LCD market grows, significant amount of rare material of indium (In) has been continuously consumed in the display manufacturing industry. Indium has been widely used for transparent conductive oxides (TCO) in the form of indium tin oxide (ITO), because it has unique high optical transparency and electrical conductivity. The In resource is quite limited, e.g., the world’s reserve is merely 16,000 tons while the current annual consumption is about 1400 tons [1]. Consequently, global In resources are being exhausted due to their fast consumption rate in information displays, solar cells and lighting [2,3]. Therefore, recycling of indium has become a very important issue these days. A lot of research works aimed at the reuse of In materials has been performed and, as an alternative, the recycling of In from old ITO targets has been studied [4]. However, only a few works aimed at the extraction of In from TFT-LCD panel scraps has been performed. Moreover, we could not find any result on recovery of ITO with the optimal composition ratio of In/Sn as 91/9, which is the characteristics composition of the TCO. Several works reported that In can be recovered by crushing TFT-LCD panel scraps followed by selective extraction. Acquisition of ITO from the TFT-LCD scrap with the optimal ratio is very valuable for cost saving by reducing the number of steps in the recovery process. In this study, a new recycling technology was developed to recover ITO nanoparticles from TFT-LCD scraps. It is called lift-off method because ITO layer is simply lifted off the glass substrate of panel scraps by dissolving color filter (CF) layer which is located between them as in Figure 1. Characteristics such as particle size, thermal property, crystal structure, composition ratio, and purity of the recovered ITO product were investigated.

## 2. Experimental Section

An alkaline solution was received from D. Company (Seoul, Korea) and used as a dissolving agent for the color filter. The solution consists of mainly KOH and NaOH and its pH is about 13.4. Polycarbonate (PC) membrane (Iso pore) having 0.4 μm pore diameter was purchased from Milllipore and used as a filter. Methylene Chloride (extra pure grade, Daejung Chemical and Metals, Korea) was used to dissolve the PC membrane. Deionized (DI) water was used to wash out the sample after each step. ITO was taken from a 7 inch size TFT-LCD panel scraps. Disassembly of the TFT-LCDs was done according to previously published methods [5]. First, the upper and lower portions were separated by cutting the edge of the TFT-LCD panel scraps. After cutting, the two plates were easily separated by removing residual liquid crystal with acetone. The upper plate was dipped into the alkaline solution at 80 °C for 30 min. Then the ITO was separated from the glass substrate automatically and remained in the solution after taking the glass substrate out of the solution. The ITO precipitate in the solution was filtered and washed several times with D.I. water. Filter cake was dried in a convection oven at 60 °C for 1 h. Then the thermal behavior of the filter cake was analyzed by thermogravimetric analysis (TGA) and differential thermal analysis (DTA) were performed by using thermogravimetry/differential thermal analyzer (TG/DTA: Seiko Exstar 6000, Seico Inst., Japan). In this way, the heat-treatment temperature to burn out the organic components was determined. The particle size, crystal structure, composition ratio, and purity of the ITO was analyzed by means of high-resolution transmission electron microscope (HRTEM: 300 kV, JEOL, Japan), field-emission scanning microscope (FESEM: JSM-7000F, JEOL, Japan), X-ray diffractometer (XRD: Rigaku Rotaflex D/MAX System) with monochromatic Cu target (λ = 0.1541 nm), inductively coupled plasma (ICP, ICP Optima 43DV, Perkin Elmer, Waltham, MA, USA), X-ray photoelectron spectroscopy (XPS: VG Microtech ESCA2000), using Al Kα radiation as an exciting source. All binding energies (BE) were referenced to the C 1s peak at 284.6 eV.

## 3. Results and Discussion

**Figure 2:**
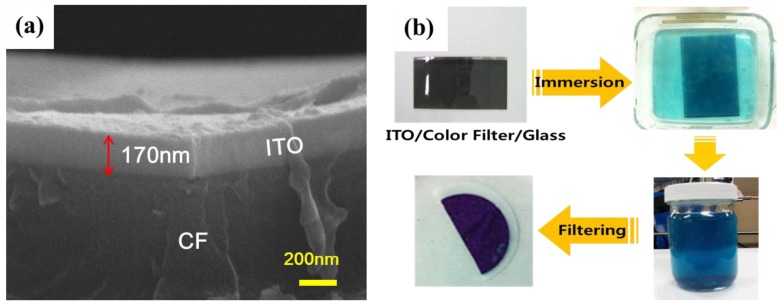
(**a**) SEM image of ITO taken from a car navigator; (**b**) schematic process for the recovery of ITO.

Figure 2a shows the cross-sectional SEM image of 7 inch LCD upper plate taken from a car navigator. The thickness of ITO was observed as 170 nm. Figure 2b shows the simple recovery scheme. The upper plate was immersed into the alkaline solution. After 30 min, the colorless alkaline solution was changed to blue color. That means some parts of RGB pigments were dissolved. This solution was filtered with the polycarbonate (PC) membrane (pore size 0.4 µm).

**Figure 3:**
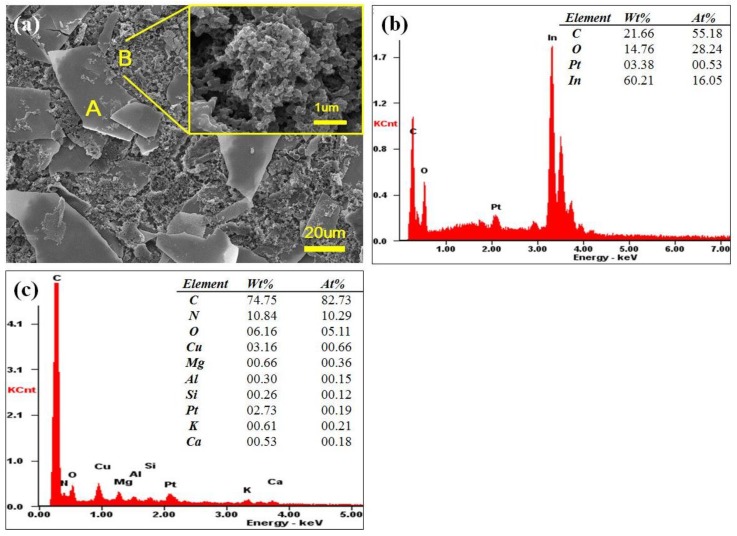
(**a**) SEM image of filter cake; EDS results for the (**b**) part A and (**c**) part B.

The filter cake appeared as two parts, film shape part and granular part as in Figure 3a. In the part A, many of irregular film pieces were found. According to the energy dispersive spectrometer (EDS) analysis as shown in Figure 3b its major component was indium. Therefore we estimated that these pieces came from ITO. The granular part B was quite different from the part A. The EDS analysis in Figure 3c shows the elemental components of the aggregate part are carbon, nitrogen, oxygen, copper and magnesium *etc.* indicating this part came from color filter.

**Figure 4:**
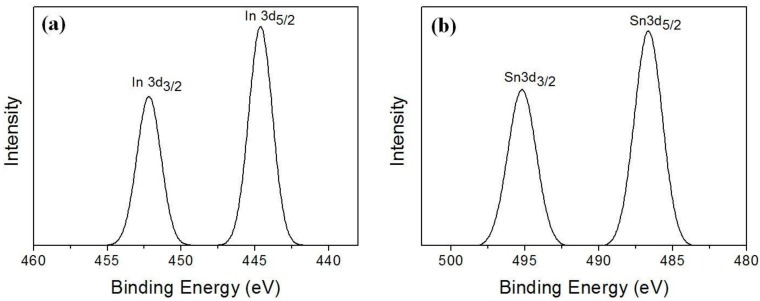
XPS spectra of filter cake part A; (**a**) Indium 3d and (**b**) Tin 3d.

The specific analysis of ITO with EDS is very difficult because energy levels of indium and tin are too close to differentiate each other. Therefore, we studied the composition via XPS measurement. The XPS spectra of filter cake are shown in Figure 4. As shown in Figure 4a the peaks located at 444.6, and 452.2 eV corresponding to the In 3d_5/2_ and In 3d_3/2_ state, respectively. These are related to In^3+^ bonding state in the In_2_O_3_ [6,7,8,9,10]. Figure 4b shows that Sn 3d_5/2_ and Sn 3d_3/2_ peaks located at 486.6, 495.2 eV respectively. These binding energies indicate that the Sn is in the Sn^4+^ bonding state from SnO_2_ [7,10]. According to the XPS analysis, the binding energy of filter cake, part A, was in good agreement with a typical ITO. Therefore, it is concluded that the recovered sample was mostly consisted of ITO.

**Figure 5:**
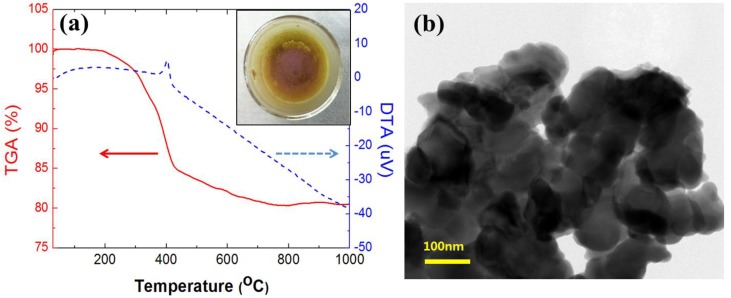
Thermal behavior (**a**) and high-resolution transmission electron microscope (HRTEM) image (**b**) of the recovered ITO powder from TFT-LCD panel after heat treatment at 600 °C.

Most of the filter cake was collected by dissolving the filter with the filter cake with methylene chloride. After decanting the methylene chloride away, the residue was washed with same solvent several times. Thermal behavior of the residue was performed by using TG/DTA. As shown in Figure 5a, TG/DTA curves were obtained in the range of ambient temperature to 1000 °C at a heating rate of 10 °C·min^−1^ in air at atmospheric pressure. The first weight loss was observed in temperature range, *i.e.*, between 200 °C and 400 °C, and DTA curve shows the exothermic reaction showing a peak at around 400 °C. This weight loss maybe due to decomposition of CF pigments in the residue. Composition of the CF materials really depends on the producing company but most of them are based on anthraquinone (red) and phtalocyanine (green or blue) [11]. These materials show the weight loss and exothermic reaction at temperatures lower than 600 °C [12,13,14]. These results indicate that thermal decomposition of the CF pigments could occur at lower than 600 °C. The inset in Figure 5a shows the yellow ITO powder obtained after heat treatment at 600 °C for 1 h in air. The color was similar to that of commercial ITO powder. The morphology of recovered ITO was observed by using HRTEM. The sizes of recovered ITO particles were less than 100 nm in diameter and appeared as aggregate as shown in Figure 5b.

**Figure 6:**
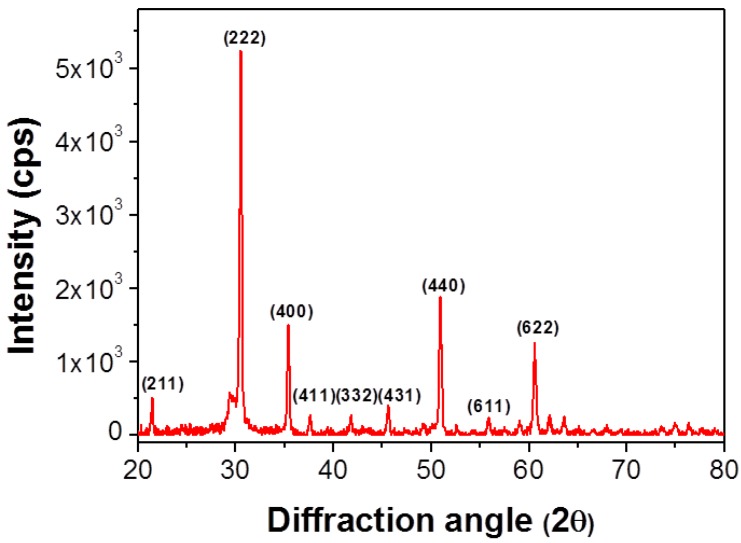
XRD pattern of ITO nanoparticles recovered after heat-treatment at 600 °C.

Figure 6 shows the X-ray diffraction pattern of the recovered ITO powder after heat-treatment at 600 °C. This pattern indicates that our sample has quite similar structure compared to a typical ITO crystal structure. The most intensive diffraction peak appeared at 30.5° and other peaks were at 35.5°, 50.9° and 60.7°. This pattern matches well with the diffraction pattern for the (222), (400), (440) and (622) orientations of cubic crystalline ITO [15,16]. Thus, the ITO particles after heat-treatment at 600 °C are well crystallized as cubic structure. However, the splitting of diffraction peak appeared at 30° indicates that the recovered ITO consists of two different layers. According to the reported results [17,18,19], splitting of XRD peak in ITO films was attributed to existence of two differently strained layers, *i.e.*, amorphous and crystalline layer. The diffraction pattern of the recovered material was similar to that of pure ITO. It is supposed that most In atoms are uniformly substituted to Sn atoms in the lattice [20].

The composition ratios of In and Sn was evaluated before and after heat treatment by using ICP analysis. In and Sn compositions before and after heat treatment were summarized in Table 1. The composition of In and Sn before heat treatment was 91.2 wt.%, 8.8 wt.%, but after heat treatment it was changed to 91.6 wt.%, 8.4 wt.%. The compositions were changed a little after heat treatment. Unfortunately, the reason is not clear up to now and is being under investigation. Nevertheless, the composition was exactly closed to 91/9 even after heat treatment at higher temperatures.

**Table 1:** Composition ratios of ITO nanoparticles obtained from TFT-LCD scraps by ICP analysis.

Elements	Before heat treatment (wt.%)	After 600 °C heat treatment (wt.%)
In	91.2	91.6
Sn	8.8	8.4

## 4. Conclusions

We have successfully recovered ITO in the form of nanoparticle from TFT-LCD panel scraps by using a simple lift-off method. This lift-off approach provided us a useful recovery method with high yield of 90%. ITO was easily separated as nanoparticles from glass substrate of TFT-LCD scarp. The size of the ITO nanoparticle was less than 100 nm, and appeared as aggregates. The recovered ITO was well crystallized to a (222) preferred orientation, and the composition ratio of In to Sn of about 91 to 9. The lift-off method described in this paper could be easily applied to the large scale recovery business dealing large size LCD panels from TV and other displays.
